# Supplementary material for: Sargachromenol Purified from Sargassum horneri Inhibits Inflammatory Responses via Activation of Nrf2/HO-1 Signaling in LPS-Stimulated Macrophages
Source: Mar Drugs. 2021 Aug 31;19(9):497. doi: 10.3390/md19090497 (PMC8466374; doi:10.3390/md19090497)
Supplement: Supplementary file 1 [file marinedrugs-19-00497-s001.zip › marinedrugs-1364265-supplementary.pdf]

Supplementary data

Sargachromenol purified from *Sargassum horneri* inhibits inflammatory responses via activation of Nrf2/HO-1 signaling in LPS-stimulated macrophages

Eui Jeong Han[1,2], Thilina.U. Jayawardena[3], Jae-Hyuk Jang[4], Ilekuttige Priyan Shanura Fernando[5], Youngheun Jee[6,7], You-Jin Jeon[3], Dae-Sung Lee[8], Jeong Min Lee[8], Mi-Jin Yim[8], Lei Wang[9], Hyun-Soo Kim[8,*], Ginnae Ahn[2,5*]

[1] Research Center for Healthcare and Biomedical Engineering, Chonnam National University, Yeosu 59626, Korea; iosu5772@naver.com (E.J.H)

[2] Department of Food Technology and Nutrition, Chonnam National University, Yeosu 59626, Republic of Korea; iosu5772@naver.com (E.J.H)

[3] Department of Marine Life Science, Jeju National University, Jeju 63243, Republic of Korea; tuduwaka@ jejunu.ac.kr (T.U.J); youjinj@jejunu.ac.kr (Y.-J)

[4] Natural Medicine Research Center Korea Research Institute of Bioscience and Biotechnology, Chungcheongbuk-do 28116, Republic of Korea; jangjh@kribb.re.kr (J-H.J)

[5] Department of Marine Bio-Food Sciences, Chonnam National University, Yeosu 59626, Republic of Korea; shanurabru@ jnu.ac.kr (I.P.S.F)

[6] Department of Veterinary Medicine and Veterinary Medical Research Institute, Jeju National University, Jeju 63243, Republic of Korea; yhjee@jejunu.ac.kr (Y.J)

[7] Interdisciplinary Graduate Program in Advanced Convergence Technology & Science, Jeju National University, Jeju 63243, Republic of Korea; yhjee@jejunu.ac.kr (Y.J)

[8] National Marine Biodiversity Institute of Korea, 75, Jangsan-ro 101 gil, Janghang-eup, Seocheon 33662, Republic of Korea; daesung@mabik.re.kr (D.-S.L); lshjm@mabik.re.kr (J.M.L); mjyim@mabik.re.kr (M.-J.Y); gustn783@mabik.re.kr (H.-S.K)

[9] College of Food Science and Engineering, Ocean University of China, Qingdao 266003, China; leiwang2021@ouc.edu.cn (L.W))

* Correspondence: gnahn@jnu.ac.kr (G.A); gustn783@mabik.re.kr (H-S.K.); Tel: +82-61-659-7213 (G.A); +82-041-950-0919 (H-S. K.)

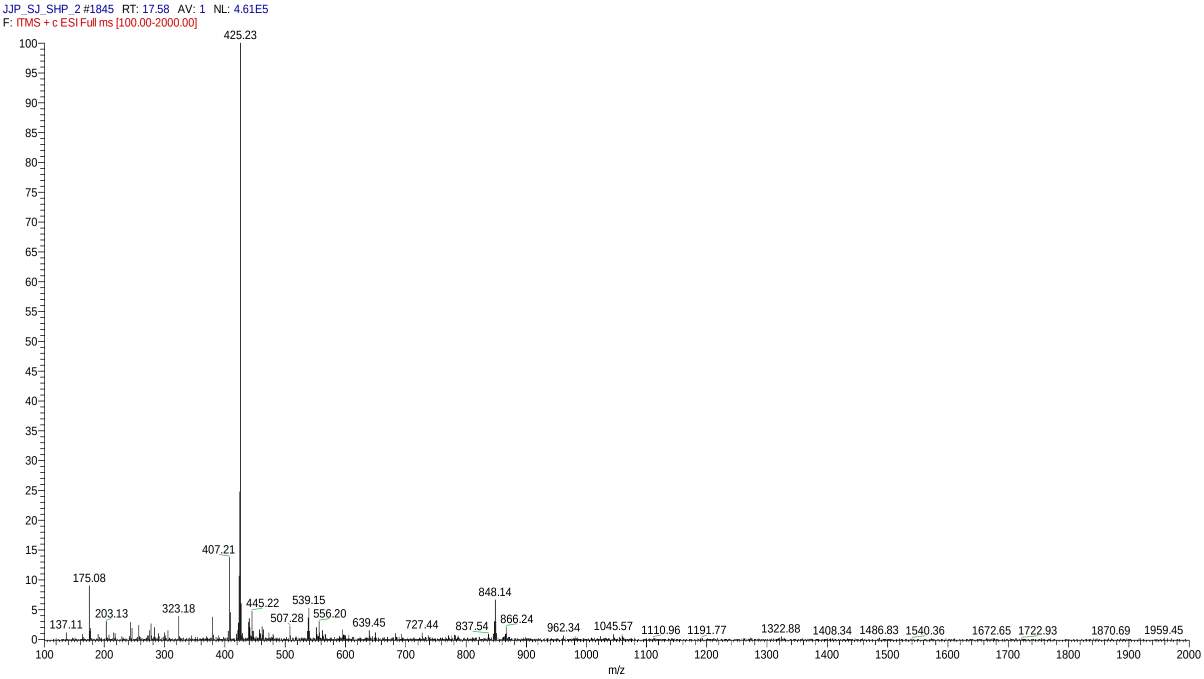

Supplemental figure S1. ESI-MS spectra (positive ion mode) of Sargachromenol.

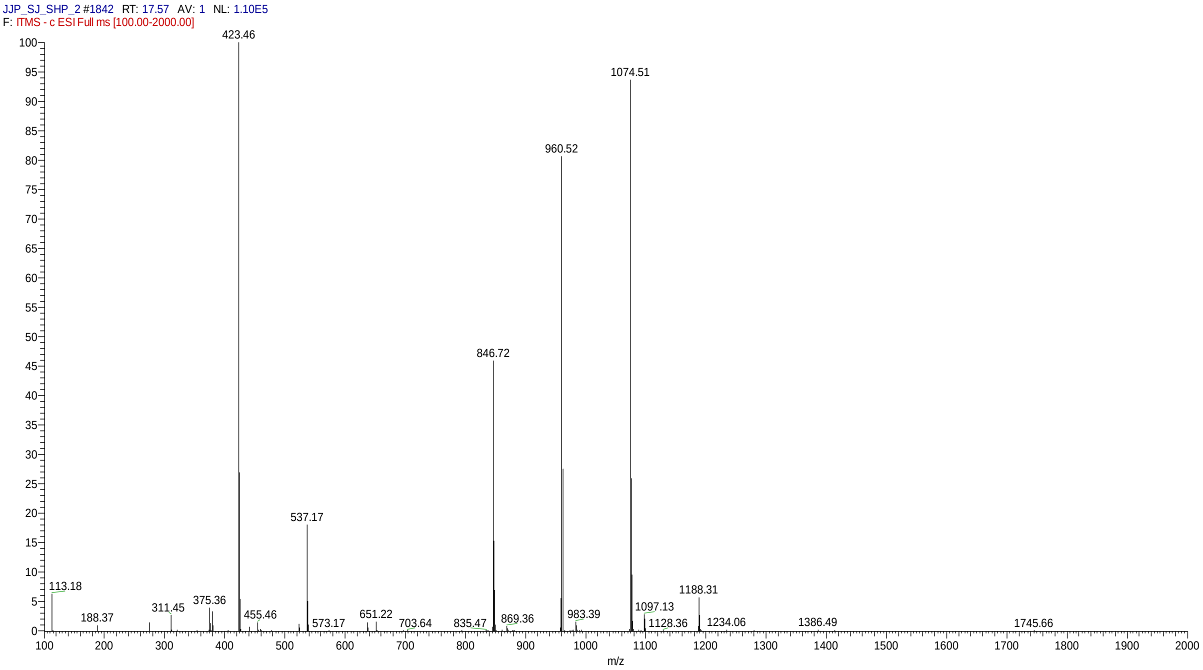

Supplemental figure S2. ESI-MS spectra (negative ion mode) of Sargachromenol
